# Complete genome sequence of *Thiovibrio* sp. Mzg5—an anaerobic chemolithoautotroph capable of thiosulfate disproportionation

**DOI:** 10.1128/mra.00710-25

**Published:** 2025-08-25

**Authors:** Kazuhiro Umezawa, Hisaya Kojima, Manabu Fukui

**Affiliations:** 1 The Institute of Low Temperature Science, Hokkaido University 12810, Sapporo, Japan; 2 The School of Food and Nutritional Sciences, University of Shizuoka 13140, Shizuoka, Japan; Wellesley College, Wellesley, Massachusetts, USA

**Keywords:** sulfur disproportionation, sulfur cycle, wastewater treatment, sulfur-disproportionating bacteria

## Abstract

The complete genome sequence was determined for a thiosulfate-disproportionating bacterium isolated from anoxic bottom water of a freshwater lake. Analyses of the 16S rRNA gene sequence and genome relatedness suggested that the strain is a close relative of *Thiovibrio frasassiensis*. Its complete genome sequence may help in understanding microbial sulfur disproportionation.

## ANNOUNCEMENT

A thiosulfate-disproportionating bacterium, strain Mzg5, was isolated from an enrichment culture previously described (1) by repeated serial dilution. The culture was originally inoculated with anoxic bottom water of a freshwater lake in Japan (Lake Mizugaki). Strain Mzg5 was isolated and routinely cultured at 28℃, with the methods for the thiosulfate-disproportionating bacterium, as described previously (2, 3). Briefly, a bicarbonate-buffered mineral medium was prepared under oxygen-free conditions to cultivate anaerobic autotrophs. The headspace of culture vessels was filled with N_2_/CO_2_ gas. The medium was supplemented with sodium thiosulfate and poorly crystalline Fe(III) oxide, as a substrate for disproportionation and a scavenger of sulfide, respectively.

Genomic DNA was extracted with the cetyltrimethylammonium bromide (CTAB) method as described previously (4), and further purified with the NucleoSpin gDNA Clean-up Kit (Takara Bio) to subject sequencing with DNBSEQ and GridION platforms. For the DNB sequencing, genomic DNA was fragmented to ca. 400 bp by using Covaris. With the fragmented DNA, a library was prepared by using MGIEasy Universal DNA Library Prep Set and MGIEasy DNA Adapters-96 (Plate) Kit (MGI), following the manufacturer’s protocols. From the PCR products, circular DNA was prepared by using the MGIEasy Circularization Kit (MGI). DNA nanoballs were prepared with MGISEQ-2000RS High-throughput Sequencing Set and sequenced with DNBSEQ-G400 (paired-end sequencing, 2 × 150 bp). From 10,852,591 reads generated, around 3,500,000 read pairs were subsampled with Seqkit ver. 0.11.0 (5), and subjected to quality filtering with Sickle ver. 1.33 (https://github.com/najoshi/sickle). For the GridION sequencing, the purified genomic DNA was ligated to barcodes using Native Barcoding Expansion (EXP-NBD104, Oxford Nanopore Technologies), without fragmentation or size selection. With the barcode-ligated DNA, sequencing library was prepared by using Ligation Sequence Kit (SQK-LSK109, Oxford Nanopore Technologies). The library was sequenced on the R9.4.1 flow cell, and basecalling was performed with Bonito. The generated 358,143 raw reads (average length = 1391.8; read length N50 = 2,636) were trimmed with Porechop ver. 0.2.3 (https://github.com/rrwick/Porechop), filtered with Filtlong ver. 0.2.0 (https://github.com/rrwick/Filtlong), and then subjected to error correction with Canu ver. 1.8 (6). The qualified and corrected reads were subjected to hybrid assembly with Unicycler ver. 0.4.7 (7) with the default settings. As a result, a single circular chromosome was generated with coverage of 460.7-fold. Its size and G + C content were 2,921,245 bp and 57.2%, respectively. In the genome annotated and rotated by DFAST ver. 1.2.4 (8), 2,700 coding sequences (CDSs), 6 rRNA genes, and 50 tRNA genes were identified. Analysis of the 16S rRNA gene sequence indicated that strain Mzg5 is the closest relative of *Thiovibrio frasassiensis* RS19-109^T^ (9), with a sequence identity of 98.7%. To determine the genome relatedness between these strains, digital DNA–DNA hybridization (dDDH) was calculated by using Genome-to-Genome Distance Calculator ver. 3.0 (10). The dDDH value calculated with the formula 2 was 25.7%.

The genome sequence reported here (AP022872) will help in understanding the mechanism of microbial sulfur disproportionation. Further studies on the strain Mzg5, deposited at DSMZ and NRBC (DSM 111103/NBRC 114533), may provide a unique opportunity to develop water treatment systems that utilize sulfur disproportionation (11–13).

## Data Availability

The genome sequence has been deposited in DDBJ under the accession number AP022872. The raw reads of DNBSEQ and GridION have been deposited in the DDBJ Sequence Read Archive (DRA) under the accession numbers DRR700366 and DRR700367, respectively. The accession numbers for BioProject and BioSample are PRJDB9418 and SAMD00209864, respectively.
